# Shape-based Automatic Detection of Pectoral Muscle Boundary in Mammograms

**DOI:** 10.1007/s40846-015-0043-6

**Published:** 2015-06-10

**Authors:** Chunxiao Chen, Gao Liu, Jing Wang, Gail Sudlow

**Affiliations:** Department of Biomedical Engineering, Nanjing University of Aeronautics and Astronautics, Nanjing, 210016 China; Department of Radiology, Washington University School of Medicine, St. Louis, MO 63110 USA

**Keywords:** Pectoral muscle, Boundary detection, Shape-based mask, Mammogram

## Abstract

The detection of the pectoral muscle boundary in the medio-lateral oblique view of mammograms is essential to improving the computer-aided diagnosis of breast cancer. In this study, a shape-based detection method is proposed for accurately extracting the boundary of the pectoral muscle in mammograms. A shape-based enhancement mask is applied to the mammogram and the initial boundary is then defined using morphological operators. The seed point is then detected on the initial boundary and the pectoral boundary is evolved from candidate points produced using a shape-based growth strategy. A cubic polynomial fitting function is implemented to obtain the final pectoral muscle boundary. The proposed method was applied to 322 mammograms from the mini Mammographic Image Analysis Society database. A 97.2 % acceptable rate from expert radiologists and assessment results based on the false positive rate, false negative rate, and Hausdorff distance demonstrate the robustness and effectiveness of the proposed shape-based detection method.

## Introduction

The three most commonly diagnosed types of cancer among women in 2013 were breast, lung, and colorectal cancers, accounting for 51 % of estimated cancer cases in women, with breast cancer alone accounting for 29 % [1]. Breast cancer is the most common cause of cancer-related deaths, and early detection is the best defense against it [2]. Compared with other detection techniques, such as magnetic resonance imaging, nuclear medicine, and ultrasound, mammography has the advantages of low er cost, higher convenience, and higher spatial resolution. However, it is challenging for physicians to correctly and quickly interpret a large number of mammograms. Computerized mammographic analysis has been proposed to improve efficiency and avoid inter-observer discrepancies [3].

In the computerized mammographic analysis, three anatomical landmarks, namely the breast border, nipple, and pectoral muscle, are first extracted automatically [4]. The present study mainly focuses on improving the accuracy of pectoral muscle extraction. The pectoral muscle, which is a predominantly dense region in most medio-lateral oblique (MLO) views, always appears as a high-intensity, triangular region across the upper posterior margin of the image and has texture characteristics similar to those of mammographic parenchyma, which can easily cause a high false positive (FP) rate and misdiagnosis of breast cancer. The pectoral edge is used as one of the axes in three-dimensional reconstructions from a large series of two-dimensional mammographic views [5]. However, the wide variability in size, intensity, shape, and position of the pectoral muscle due to the individual difference and patient positioning during image acquisition, together with the similarity between muscle and breast tissues, make pectoral muscle detection very challenging [6].

In this study, shape-based detection is proposed to automatically segment the pectoral muscle boundary. A shape-based enhancement mask (SBEM) is first implemented to highlight the pectoral muscle boundary, and then the pectoral muscle boundary is evolved using a seed point and a shaped-based search strategy. The rest of this paper is organized as follows. Works related to pectoral muscle extraction are reviewed in Sect. 2. Section 3 described the proposed automatic detection method of the pectoral muscle boundary in mammograms. The experimental results and discussion are given in Sects. 4 and 5, respectively. Section 6 gives the conclusions.

## Related Works

Several studies have proposed methods for pectoral muscle segmentation. On the assumption that the pectoral muscle boundary is approximate to a straight line at an angle of between 45° and 90°, the Hough transform was applied to extract the edge as a line [6]. The parenchymal pattern classification was applied to 615 oblique mammograms and 65 % of the results were consistent with the results of radiologists, but the performance of pectoral muscle identification was not reported. Kwok et al. [7] presented straight line estimation and iterative cliff detection methods to identify the pectoral muscle. The detection rate validated by two expert mammographic radiologists was 83.9 % for the mini Mammographic Image Analysis Society (mini-MIAS, http://peipa.essex.ac.uk/ipa/pix/mias/) database of 322 images. The Radon transform was applied to automatically detect the straight line approximating the edge of the pectoral muscle [8]. 540 MLO mammograms from the Medical Center of the Faculty of Medicine, University of São Paulo, were tested. 69.6 % of the detection results agreed with a radiologist’s results. To solve the problem of detecting a non-linear pectoral muscle boundary, the Gabor wavelet filter was applied to enhance the pectoral muscle boundary for segmentation [9]. This approach overcomes the limitation of a straight-line representation, but often fails when the interface of the glandular tissue and the pectoral muscle is not very clear. For 84 MLO mammograms from the mini-MIAS database, the Gabor wavelet method achieved average FP and false negative (FN) rates of 0.58 and 5.77 %, respectively. Various methods based on the intensity differences between the breast tissue and the pectoral muscle have been proposed to extract the pectoral muscle boundary, such as the region growth technique [10], the intensity cliff detection algorithm [11], and the gradient-based texture analysis method [12]. However, to some extent, the validity of these methods is greatly affected by the intensity contrast between pectoral muscle and breast tissues. Two methods based on graph theory have been proposed for identifying the pectoral muscle [13]. For 84 MLO mammograms from the mini-MIAS database, a graph-pectoral-segment method based on adaptive pyramids (AP) obtained average FP and FN rates of 3.71 and 5.95 % and a method based on minimum spanning trees (MST) achieved average FP and FN rates of 2.55 and 11.68 %, respectively. The watershed transformation (WaT), a commonly used segmentation technique, has been applied to extract the pectoral muscle [14]. The mean FP and FN rates were 0.85 and 4.88 %, but over-segmentation was difficult to avoid. Additionally, various enhancement approaches for mammograms are commonly adopted prior to extracting the boundary of the pectoral muscle. Charkraborty [15] designed a weight function to highlight the boundary, and then utilized the local gradient to find the edge points; however, the FP pixel percentage was ≥4.22 %. A combination of adaptive histogram equalization and polynomial curvature estimation on the selected region of interest was implemented to enhance the contrast of mammograms, which makes the segmentation of very-low-contrast pectoral muscle areas possible; 96.56 % of the test results were acceptable [16]. Li [17] employed two features of the pectoral muscle, namely homogeneous texture and high-intensity deviation (HT-HID), to identify the initial pectoral muscle edge, and then used the Kalman filter to refine the ragged initial edge. The acceptable segmentation result rate was 90.06 % for the mini-MIAS database. The definition of acceptable segmentation result rate is given in the Results section.

## Methods

**Fig. 1:**
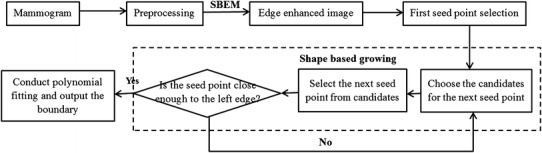
Illustration of pectoral muscle detection

The pectoral muscle has significant anatomical features, such as sharp intensity changes on the boundary, roughly triangular shape, and gradually narrowing from top to bottom [17]. Based on these characteristics, an SBEM and a boundary evolution strategy are proposed in this paper to automatically detect the pectoral muscle boundary, as shown in Fig. 1.

### Shape-based Enhancement Mask

**Fig. 2:**
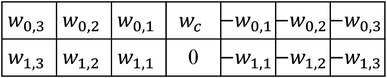
Linear enhancement mask

**Fig. 3:**
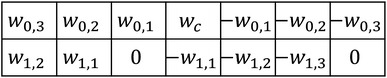
Shape-based enhancement mask

The pectoral muscle usually has an approximate direction and higher gray level intensity in mammogram. Based on the prior knowledge, an enhancement filter is commonly used to process mammograms to highlight the pectoral muscle. Zhou et al. [12] developed a gradient-based directional kernel (GDK) filter to enhance the linear texture structures on mammograms at approximately 45° from the top left to the bottom right and implemented it by convolving the image with an 11 × 11 mask with values of 1 and −1. However, the GDK filter is very sensitive to the ridge points and produces a lot of unwanted boundaries. To overcome these problems, a linear shape-based enhanced filter with several coefficients that considers the transition intensity changes around the pectoral muscle edge is proposed here. The filter is designed as:

1 $$g(x,y) = w_{s,t} \sum\limits_{s = 0}^{M} {\sum\limits_{t = 1}^{N} {(I(x + s,y - t) - I(x + s,y + t)) + w_{c} I(x,y)} }$$

where $I(x,y)$ is the intensity of the pixel at point (*x*, *y*), (*M* + 1) is the number of rows, $N$ is the number of pixel pairs contributing to the weighted differentiation along the horizontal direction, and $w_{s,t}$ and $w_{c}$ are weight coefficients. The expression can be implemented by convolving a mammogram with a linear enhancement mask (Fig. 2). Considering that the pectoral muscle gradually narrows from top to bottom, the bottom coefficients of the mask are shifted to the left to highlight the structural characteristics, as shown in Fig. 3. Due to the pixel intensity gradually becoming stronger away from the left side of the boundary, in this mask, the coefficient $w_{s,t}$ increases with $t$ to suppress unwanted tissues, and the diagonal coefficients enhance the structural characteristics of the pectoral muscle. The sum of the mask coefficients (excluding $w_{c}$) is zero. In the filtered image, the regions with homogenous intensities in the original image are suppressed and the boundary is emphasized. The coefficient $w_{c}$, which represents the contribution of the center point, is often set in the range of 0–1 to avoid excessive influence on filter results while processing non-boundary regions with a high intensity value.

### Seed Point Selection

Based on the characteristics of the pectoral muscle, Domingues [18] predicted two endpoints of the pectoral muscle and then computed the muscle contour using a shortest path technique. However, in many cases, the endpoint detection of the contour on the left column is difficult to precisely implement since the lower half of the pectoral muscle is often invaded by glandular tissues, which seriously affects the extraction of the shortest path. Here, a method for selecting the start point of the pectoral muscle on the boundary is proposed to facilitate obtaining the true boundary of the pectoral muscle. In order to avoid the confusion caused by glandular tissue, the start point (seed point) of the boundary is searched for only on the top part of the enhanced image. The start point search strategy is as follows:

Step 1 Define $P$ row pixels on the top part of the enhanced image as the search subimage.
Step 2 Threshold the subimage, keeping $Q$ largest values for each row.
Step 3 Morphological operators with size of three are used to detect the edges in the subimage.
Step 4 Define the initial boundary of the pectoral muscle. Edges with an angle of less than 90° along the horizontal direction are selected, and the number of pixels of each edge is counted. The edge with the largest number of pixels is then defined as the initial boundary of the pectoral muscle. When two or more edges have almost the same number of pixels, the edge with the highest average intensity value is defined as the initial boundary. Furthermore, if the average intensity values of these edges are similar, this pectoral muscle is considered as consisting of multiple layers and the edge on the right is defined as the initial boundary.
Step 5 The top right point on the initial boundary is defined as the seed point for pectoral muscle boundary growth.

### Shape-based Growth Strategy

**Fig. 4:**
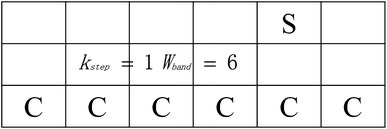
Shape-based growth strategy

The pectoral muscle boundary is often obtained by refining a straight line using intensity information [7, 15]. However, the estimated straight line seriously affects the extraction accuracy of the pectoral muscle boundary. In this study, to get an accurate boundary of the pectoral muscle, a simple and convenient boundary detection method based on the start point is proposed to segment the pectoral muscle in a mammogram. Based on the characteristics of the pectoral muscle, a shape-based growth mask is designed as shown in Fig. 4, in which S and C represent the current seed point and candidate point, respectively. The number of candidates is $W_{band}$ and the row interval is$K_{step}$. The proper selection of $K_{step}$ can reduce the effects of noise and fibro-glandular tissues. Different from traditional region growth methods, most of the candidates are placed on the left side of the current seed point to match the shape of the pectoral muscle, which gradually narrows from top to bottom. The start point is defined as the first seed point. The candidate point with the maximum value is then selected as the next seed point. The process is iterated until the new seed point is close enough to the left side of the image. All seed points produced by the shape-based growth mask are fitted by a cubic polynomial function to create a boundary.

### Quantitative Evaluation

In order to quantitatively evaluate the accuracy of the proposed method, the FP rate, FN rate, and Hausdorff distance [19] are used. FP pixels are defined as pixels in the detected pectoral region but not in the ground truth region. FN pixels are defined as pixels in the ground truth pectoral region but not in the detected region. The FP pixel rate, FN pixel rate, and total mismatched pixel rate are respectively computed as:

2 $${\text{FP pixel rate}} = \frac{{\left| {{\text{D}} \cup {\text{R}}} \right| - \left| {\text{R}} \right|}}{{\left| {\text{R}} \right|}} \times 100\,\%$$

3 $${\text{FN pixel rate}} = \frac{{\left| {{\text{D}} \cup {\text{R}}} \right| - \left| D \right|}}{\left| R \right|} \times 100\,\%$$

4 $${\text{Total mismatched pixel rate}} = \frac{{2 * \left| {{\text{D}} \cup {\text{R}}} \right| - (\left| D \right| + \left| R \right|)}}{\left| R \right|} \times 100\,\%$$

where $D$ is the set of pixels in the detected pectoral muscle region and $R$ is the set of pixels belonging to the ground truth pectoral muscle region.

The Hausdorff distance is used to determine the similarity between identified point set and the ground truth set. It is defined as:

5 $${\text{H(A,\,B) = max((h(A,\,B),\,h(B,\,A)),\,h(A,\,B)}} = \mathop {\hbox{max} }\limits_{{{\text{a}} \in {\text{A}}}} \mathop {\hbox{min} }\limits_{{{\text{b}} \in {\text{B}}}} \left\| {{\text{a}} - {\text{b}}} \right\|$$

where $A$ is the set of detected-boundary points and $B$ is the set of ground truth boundary points. $\left\| \cdot \right\|$ is the Euclidean distance between points $a$ and $b$.

## Results

The proposed method of pectoral muscle segmentation was tested using the digitized mammograms from the mini-MIAS database. The mini-MIAS dataset contains 322 mammograms with a size of 1024 × 1024 pixels and 8 bits per pixel. Each mammogram was obtained from the MLO view and digitized with a spatial resolution of 200 µm. All mammograms were downsampled by a factor of 2, and flipped to make the pectoral muscle located on the top left side. The detection procedure of the pectoral muscle took approximately 0.3 s using a computer with a Pentium Dual-Core 2.6-GHz CPU and 4 GB of RAM in a Matlab 2012b environment.

**Fig. 5:**
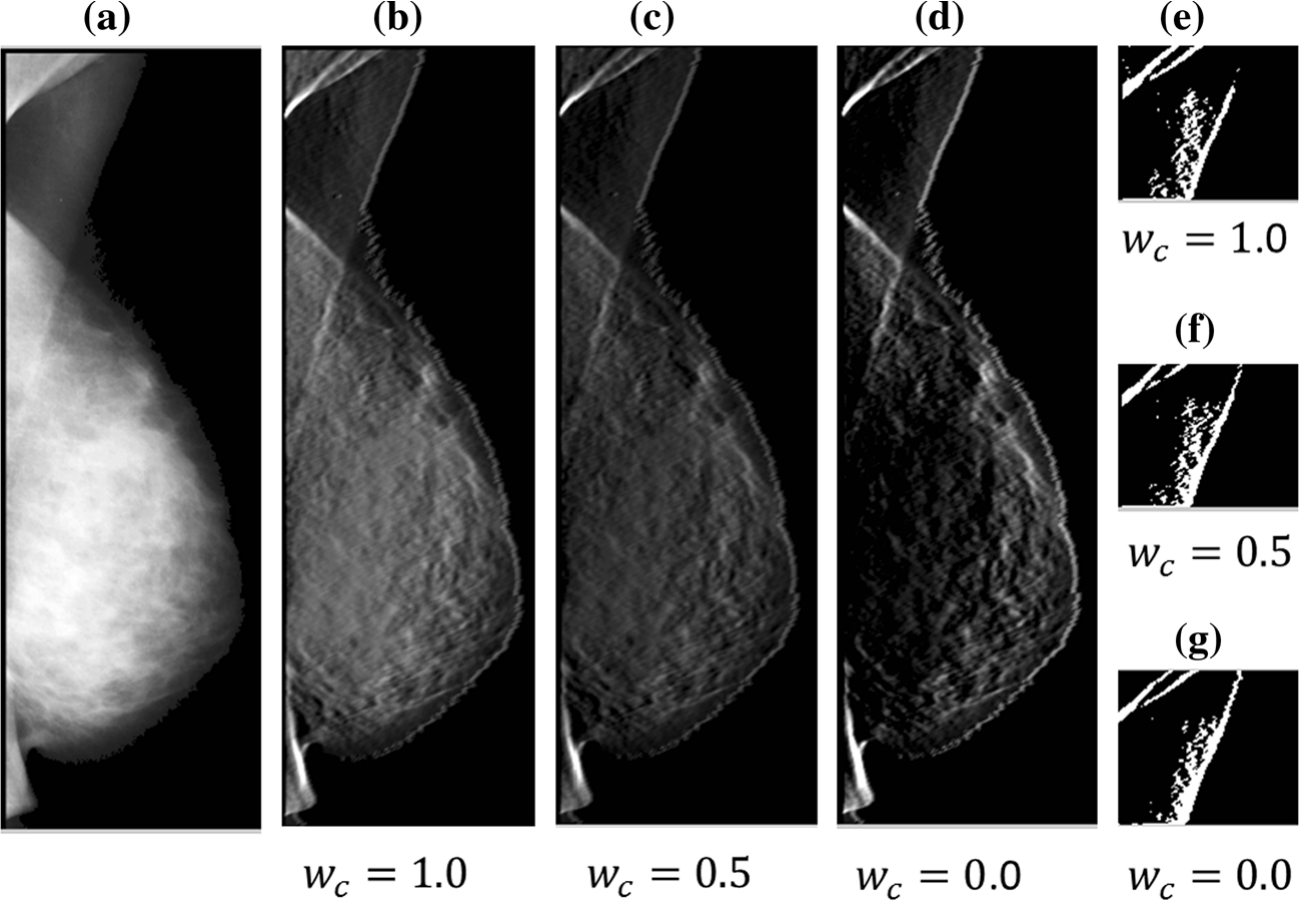
**a** Mammogram mdb065 and results of **b**–**d** gradient image and **e**–**g** edges. $w_{c}$ is 1.0, 0.5, and 0.0, respectively

322 mammograms were processed using the SBEM. Table 1 lists the parameters used in this study. Parameters$w_{0,1}$ and $w_{1,1}$ were set to 1, and $w_{1,2}$ and $w_{2,2}$ were set to 2 to increase the suppression of tissues away from the boundary. In order to keep the sum of coefficients (excluding $w_{c}$) at zero, $w_{0,3}$ and $w_{1,3}$ were set to zero in the shifted enhancement mask. Parameter $w_{c}$ represents the current pixel’s intensity contribution. Normally, a larger value of $w_{c}$ leads to better highlighting of the pectoral muscle boundary. However, as a mammogram has multiple layers and the intensities of the inside layers are much stronger than that of the outside layer (Fig. 5a), a shape-based mask sometimes cannot enhance the real edge of the pectoral muscle. Figures 5(b–d) show enhanced images with various values of $w_{c}$, and Fig. 5(e–g) show the detected edges with $P$  = 100, $Q$  = 12, and various $w_{c}$ values. In order to effectively enhance the boundary of the outside layer, as a compromise, $w_{c}$ is generally set to 0.5. $P$ and $W_{band}$ depend on the size and spatial resolution of the image. $Q$ and $K_{step}$ are determined from experiments. The proper selection of $K_{step}$ can reduce the interference introduced by noise and fibro-glandular tissue.

**Table 1:** Parameters used in pectoral muscle boundary detection

Parameter	$w_{0,1}$	$w_{0,2}$	$w_{0,3}$	$w_{1,1}$	$w_{1,2}$	$w_{1,3}$	$w_{c}$	$P$	$Q$	$K_{step}$	$W_{band}$
Value	1	2	0	1	2	0	0.5	100	12	2	6

**Fig. 6:**
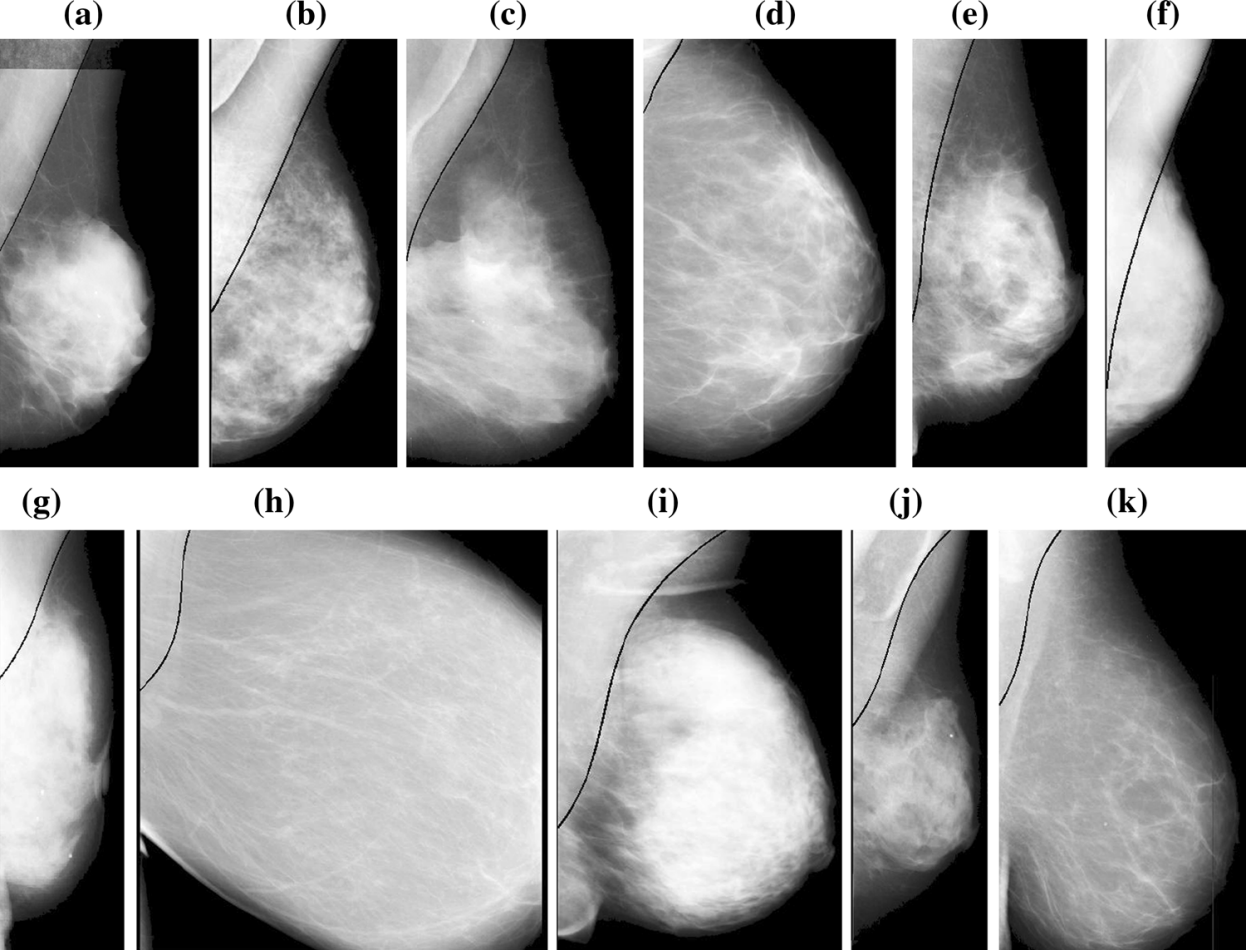
Pectoral muscle detection results of MLO mammograms **a** mdb002, **b** mdb123, **c** mdb110, **d** mdb050, **e** mdb225, **f** mdb053, **g** mdb288, **h** mdb151, **i** mdb240, **j** mdb223, and **k** mdb183

Some representative pectoral muscle detection results of MLO mammograms are shown in Fig. 6. To evaluate the quality of the pectoral muscle detection method, the boundaries of each image were manually drawn by the author, and checked by two radiologists individually. When differences existed, a consensus was reached after discussion. These manual contours were then used as the ground truth. The boundary extraction results were classified into three categories: successful, acceptable, and unacceptable. For successful results, the detected boundary was identical to the manual one. For acceptable results, more than half of the muscle boundary was correct, with and only limited discrepancy for the lower half part. All other results were unacceptable. Table 2 lists the detection results for the 322 mammograms from the mini-MIAS database. 97.2 % of the results were successful or acceptable. Furthermore, 84 mammograms used by Ferrari [9] were selected for quantitative evaluation. The mean FP and FN rates were 1.02 and 5.63 %, respectively, and the mean and standard deviation of the Hausdorff distance were 3.53 and 1.61, respectively. The FP, FN, and Hausorff distance values for various methods are compared in Table 3.

**Table 2:** Classification results of boundary detection for mini-MIAS mammograms

Category	Number of images	Percentage (%)
Successful	288	89.44
Acceptable	25	7.76
Unacceptable	9	2.80

**Table 3:** Pectoral muscle detection performance for various methods

Method	Hough	Gabor	AP	HT-HID	Proposed
FP (mean)	1.98	0.58	3.71	1.45	1.02
FN (mean)	25.19	5.77	5.95	5.52	5.63
FP < 5 % and FN < 5 %	11.90 %	53.57 %	59.52 %	57.14 %	58.33 %
min (FP, FN) < %5 and 5 % < max(FP, FN) < 10 %	0	0	21.43 %	32.14 %	35.71 %
min (FP, FN) < 5 % and max(FP, FN) > 10 %	0	0	13.10 %	8.33 %	4.76 %
5 % < FP < 10 % and 5 % < FN < 10 %	9.52 %	26.19 %	0	0	0
5 % < min(FP, FN) < 10 % and max (FP, FN) > 10 %	0	0	5.95 %	1.12 %	1.12 %
FP > 10 % and FN > 10 %	78.57 %	20.24 %	0	0	0
Hausdorff distance (mm)	7.08 ± 5.26	3.84 ± 1.73	NA	3.68 ± 1.50	3.53 ± 1.61

Data not provided in the publication are marked as NA

## Discussion

In the proposed pectoral muscle detection method, the shape feature and local intensity information are needed as prior knowledge. The shape feature is that the pectoral muscle is a roughly triangular region occupying a corner of a mammogram with an approximate direction. The local information of the pectoral muscle is its relatively high gray level intensity and high gradient at the edge pixels. Based on these characteristics, the shape-based method combines the intensity-based approach and region growth technique for pectoral muscle detection.

A mask with various coefficients was first designed to enhance the edges of the pectoral muscle. Compared with traditional enhancement filter masks, the shape-based mask not only considers the direction, but also takes into account transition intensity changes around the pectoral muscle edge. An accurate boundary of the pectoral muscle is still difficult to identify in a mammogram since it is disrupted by other line structures. Therefore, a search method was proposed to define the start point in the top row of the pectoral muscle. A constraint growth strategy is then used to obtain the line.

**Fig. 7:**
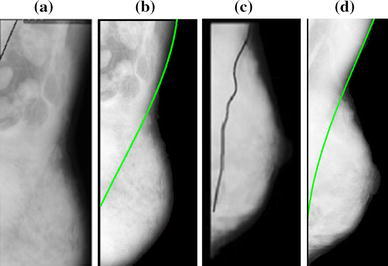
Pectoral muscle edges obtained using various methods. **a** Failed detection of mdb061 by Kwok’s method, **b** correct detection of mdb061 by proposed method, **c** inaccurate detection of mdb053 by Chakraborty’s method, and **d** accurate detection of mdb053 by proposed method

The horizontal pixels around the currently processed center are considered more in the SBEM with a 7 × 2 pixel kernel. Therefore, the pectoral muscles obscured by sticky tape can be well detected, as shown in Fig. 6a, since the intensity changes still exist in the horizontal orientation. Sometimes pectoral muscle has several layers and the inside lines can easily confuse the detection of the true edge. In the proposed method, the initial boundary is thus selected based on the prior knowledge of the pectoral muscle’s relatively high gray intensity level and location. In the segmented initial boundaries acquired from a mammogram consisting of multiple layers, the average intensity value of each layer is calculated. If these average intensity values are roughly equal, the right layer is chosen as the initial boundary based on which start point is defined. Otherwise, the edge with the highest average intensity value is regarded as the initial boundary for finding the start point. Satisfactory results (Figs. 6(b, c) are obtained using this method. The proposed shape-based growth strategy has some strong advantages. The detection results are not affected by the size of the pectoral muscle (small (Fig. 6d) or large (Fig. 6e), since pectoral muscle boundary growth depends greatly on the start point and stops when the end condition is satisfied. The upper edge also extends smoothly to the left according to the candidates designed in the shape-based growth mask, which reduces the disturbance of the dense tissue on the lower half of the pectoral muscle, producing well segmented edges (Fig. 6f, g). Furthermore, the shape-based growth mask does not set a fixed shape of the edge for growth. The edge grows well from the start point whether the pectoral muscle edge is similar to a vertical line (Fig. 6h) or is a fuzzy texture with complex curvature (Fig. 6i). However, during the initial boundary detection using the proposed method, some cases fail when the pectoral muscle has more than two layers and the inner layers have higher intensities than that of the surface layer. For these cases, the inner line would be chosen as the initial edge. The extraction of the edge in Fig. 6(j) (mdb 223) is poor because the start point on the acquired initial boundary is on the second layer of the pectoral muscle. When the upper part of the pectoral muscle is covered by other tissues and no obvious start point exists, invalid results are often obtained (Fig. 6k) (mdb 183). Figure 7 compares the proposed method and existing methods. Because the subimages are different, the fields of view have some differences. Figure 7(a) displays failed detection of mdb061 processed by Kwok and Fig. 7(c) is the inaccurate detection of mdb053 published by Chakraborty respectively. Figures 7(b,d) show the correct edges obtained using the proposed method.

Table 3 shows a performance comparison of the proposed shape-based method and methods based on the Hough transform [9], Gabor filter [9], AP [13], and HTID [17]. To some extent, the FP rate is more important than the FN rate. The shaped-based method has a good performance in terms of the mean FP rate (1.02) which is only higher than the Gabor filter result (0.58). But the detection result of the Gabor filter with both FP > 10 % and FN > 10 % is more than 20 %, which is much higher than the proposed method. Comparing with the AP method, the proposed method achieved better performance in mean FP and FN rates. And the percentage of detection results with high error term (5 % < min(FP, FN) < 10 % and max (FP, FN) > 10 %) is also much lower than AP method. As for the Hausdorff distance, the proposed method obtains the lowest mean value (3.53 mm). These results indicate that the proposed shape-based method has great performance in the extraction of the pectoral muscle boundary. It is well known that algorithm performance is related to the mammogram dataset to some degree. Therefore, the method needs to be further verified using other datasets.

## Conclusion

This study proposed an automatic method for the boundary detection of the pectoral muscle. Unlike existing methods, the proposed approach does not directly depend on region of interest or straight line detection. First, a special mask designed based on the pectoral muscle features is used to effectively enhance the boundary of mammograms. Then, an accurate start point of the boundary is determined. Based on the start point, a shape-based growth strategy is used to obtain the edge points of the pectoral muscle. Finally, a polynomial fitting function is used to determine the edge of the pectoral muscle. This method was tested on 322 digitized mammograms from the mini-MIAS database. In the future, the proposed method will be tested on mammograms from other databases, such as DDSM, to further prove its validity.
